# Aqua­{μ-1,4-bis­[(1,4,7,10-tetra­aza­cyclo­dodecan-1-yl)meth­yl]benzene}(nitrato-κ*O*)dicopper(II) tris­(nitrate) trihydrate

**DOI:** 10.1107/S2414314623004625

**Published:** 2023-06-02

**Authors:** Yoshimi Ichimaru, Koichi Kato, Kirara Sugiura, Sarina Ogawa, Wanchun Jin, Masaaki Kurihara, Yoshihiro Yamaguchi, Masanori Imai, Hiromasa Kurosaki

**Affiliations:** a Faculty of Pharmaceutical Sciences, Shonan University of Medical Sciences, 16-48, Kamishinano, Totsuka-ku, Yokohama, Kanagawa 244-0806, Japan; b College of Pharmacy, Kinjo Gakuin University, 2-1723 Omori, Nagoya 463-8521, Japan; c Environmental Safety Center, Kumamoto University, 39-1 Kurokami 2-Chome, Chuo-ku, Kumamoto, 860-8555, Japan; Purdue University, USA

**Keywords:** crystal structure, copper(II) complex, cyclen, *p*-xylene, dinuclear complex

## Abstract

In the title dinuclear Cu^II^ complex, [Cu_2_(NO_3_)(C_24_H_46_N_8_)(H_2_O)](NO_3_)_3_·3H_2_O, the two Cu^II^ mol­ecules both have a square-pyramidal geometry, but the ligands in the axial positions are different: a water mol­ecule and a nitrate ion. All nitrate ions, water mol­ecules, and N—H groups are involved in an inter­molecular hydrogen-bond network.

## Abstract 2

The title dinuclear Cu^II^ complex, the two Cu^II^ mol­ecules both have a five-coordinate square-pyramidal geometry, but the mol­ecules in the axial positions are different: a water mol­ecule and a nitrate ion. All nitrate ions, water mol­ecules, and N—H groups are involved in an inter­molecular hydrogen-bond network.

## Graphical abstract

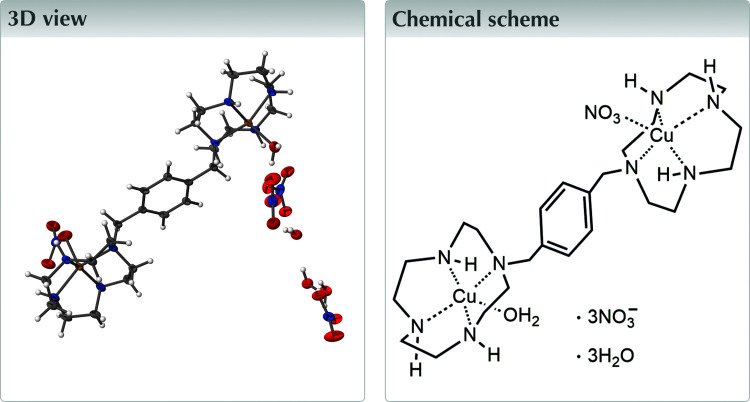

## Structure description

Cyclen (1,4,7,10-tetra­aza­cyclo­dodeca­ne) is a widely utilized macrocyclic polyamine and a water-soluble tetra­dentate ligand that can strongly chelate transition-metal cations such as Zn^II^ ions (Ichimaru *et al.*, 2021 ▸). Herein, we focused on synthesizing a ligand, *p*-bis­(cyclen), in which two cyclen rings are bridged *via p*-xylen. The dizinc complex of *p*-bis­(cyclen) has been reported as an anion acceptor for biologically active mol­ecules such as barbital (Koike *et al.*, 1996 ▸). Furthermore, the crystal structure of the perchlorate hydrate, [*p*-bis­(Cu^II^–cyclen)(ClO_4_)_4_]·4H_2_O, which is a Cu^II^ complex of *p*-bis­(cyclen), has been reported (Soibinet *et al.*, 2003 ▸).

**Figure 1:**
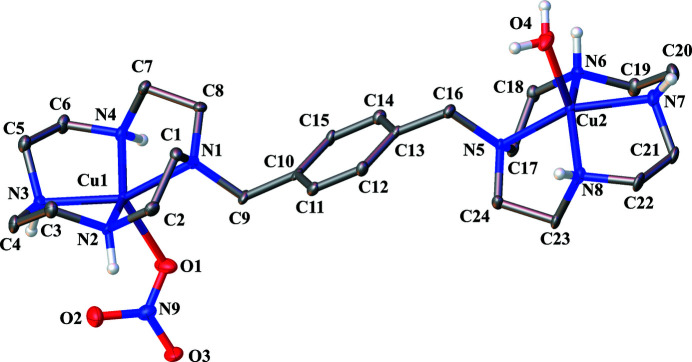
The molecular structure of the complex cation in the title compound with displacement ellipsoids drawn at the 30% probability level. C-bound H atoms, counter-anions and solvate mol­ecules are omitted for clarity.

In this context, we have prepared the nitrate salt of *p*-bis­(Cu^II^–cyclen), [*p*-bis(Cu^II^–cyclen)(OH_2_)(NO_3_)](NO_3_)_3_·3H_2_O, comprising a dinuclear Cu^II^ complex, three nitrate ions, and three water mol­ecules as the solvent (Fig. 1 ▸). Each Cu^II^ atom is five-coord­inate environment, with four nitro­gen atoms of the cyclen ring and a nitrate ion or a water mol­ecule as ligands. In the *mer*-fashion arrangement, Cu1 is coordinated by the cyclen ring (composed of N1, N2, N3, and N4) at the equatorial positions and by the nitrate ion at the axial position. Meanwhile, Cu2 is coordinated by another cyclen ring (composed of N5, N6, N7, and N8) bridged *via p*-xylene. Unlike Cu1, the axial position of the coordination polyhedron around Cu2 is occupied by a water mol­ecule. The intra­molecular Cu⋯Cu distance is close to the maximum possible value allowed by the ligand because Cu1 and Cu2 are located on opposite sides of the planar xylene spacer. Therefore, the apex of the square pyramid with Cu1 at the center points in the direction opposite to that having Cu2 at the center. The distances between Cu1 and N range from 2.011 (5) to 2.065 (5) Å, while those of Cu2 range from 2.000 (5) to 2.044 (5) Å, which are well within the typical ranges for C—N coordination bonds with amines. Soibinet and co-workers reported that the chelating nature of cyclen rings to Cu^II^ in perchlorate salts were similar to nitrate salts (Soibinet *et al.*, 2003 ▸). However, Cu^II^ is coordinated by water mol­ecules at the axial positions in both cases.

The coordination geometry index *τ* was calculated to determine the deviation from ideal coordination polyhedra around the copper ions using the formula *τ* = (*β* − *α*)/60°, where *β* and *α* are the largest and second-largest angles in the coordination center, respectively (Addison *et al.*, 1984 ▸). An ideal square pyramid has a *τ* value of 0, while an ideal trigonal bipyramid has a *τ* value of 1. The bond angles *α* and *β* of the N—Cu^II^—N chelate are 148.5 (2) and 152.0 (2)°, respectively, around Cu1 and 148.7 (2) and 153.9 (2)°, respectively, around Cu2. Accordingly, the *τ* values for Cu1 and Cu2 were calculated as 0.058 and 0.087, respectively. Therefore, the coordination geometry around the central Cu^II^ could be characterized as a marginally distorted square pyramid. The deviations from an ideal square-pyramidal geometry in certain complexes arise from the distortion of the cyclen ring (*i.e.*, the 12-membered macrocycle). Cyclam (1,4,8,11-tetra­aza­cyclo­tetra­deca­ne), a 14-membered macrocyclic polyamine, exhibits an ideal square-pyramidal environment with the transition-metal ions located in the plane formed by the nitro­gen atoms of the ring (Ichimaru *et al.*, 2022 ▸). In the title complex, Cu1 and Cu2 are located at distances of 0.521 (3) and 0.501 (3) Å, respectively, above the basal plane formed by the four nitro­gen atoms of the cyclen ring.

**Figure 2:**
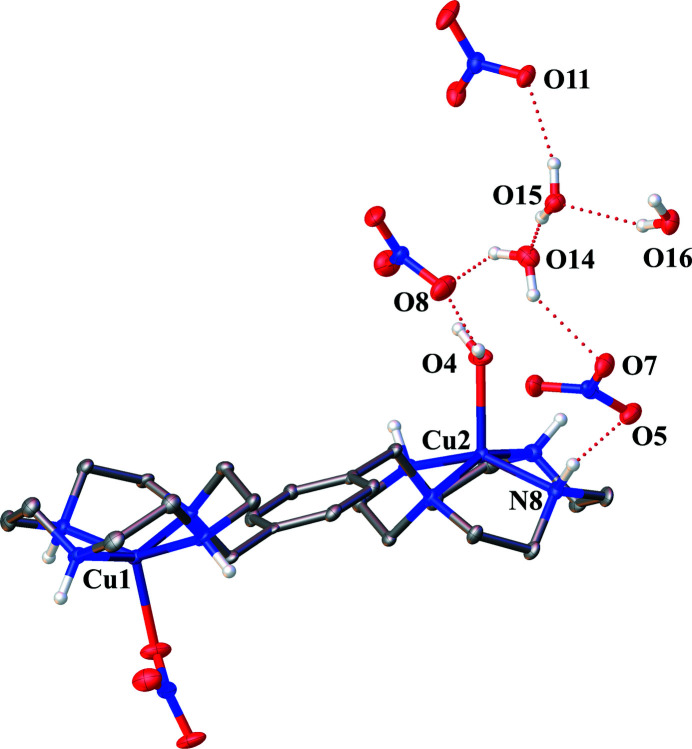
The hydrogen-bond network of the nitrate salt of *p*-bis­(Cu^II^–cyclen) with displacement ellipsoids drawn at the 30% probability level. C-bound H atoms are omitted for clarity. Hydrogen-bond inter­actions are shown as dotted lines.

All of the non-coordinating nitrate ions, water mol­ecules, and N–H groups are involved in an inter­molecular hydrogen-bond network (Fig. 2 ▸). The hydrogen bonds between the N—H groups, except N7—H7, and nitrate ions produce a hydrogen-bond network wherein water mol­ecules of solvation fill the gaps between the nitrate ions. Numerical values of the hydrogen-bonding interactions are summarized in Table 1 ▸.

**Figure 3:**
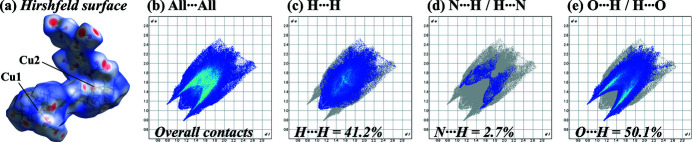
The Hirshfeld surface and two-dimensional fingerprint plots [*d*_e_ (vertical axis) and *d*_i_ (horizontal axis) represent the distances from a point on the Hirshfeld surface to the nearest atoms outside (external) and inside (inter­nal) the surface, respectively] of the nitrate salt of *p*-bis­(Cu^II^–cyclen). (*a*) Hirshfeld surface and (*b*) all inter­actions and those delineated into (*c*) H⋯H, (*d*) N⋯H/H⋯N, and (*e*) O⋯H/H⋯O inter­actions.

**Figure 4:**
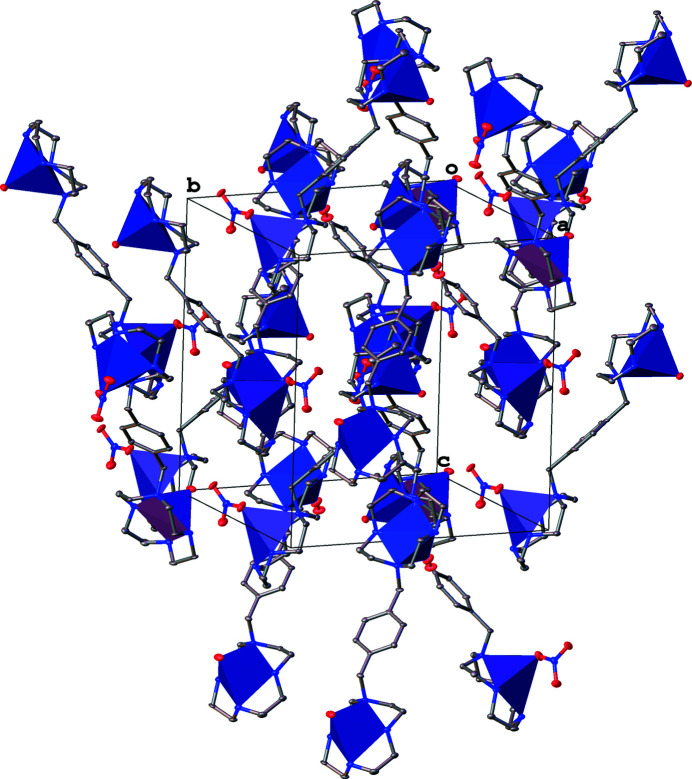
Packing view of the nitrate salt of *p*-bis­(Cu^II^–cyclen) represented with polyhedral structures around the Cu^II^ atoms, with displacement ellipsoids drawn at the 30% probability level. Non-coordinating nitrate ions, solvate water mol­ecules and C-bound H atoms are omitted for clarity.

*Crystal Explorer 21.5* (Spackman *et al.*, 2021 ▸) was used to perform a Hirshfeld surface analysis (Spackman & Jayatilaka, 2009 ▸) and generate the associated two-dimensional fingerprint plots. The Hirshfeld surface mapped over *d*_norm_ with a standard resolution is illustrated in Fig. 3 ▸ along with fingerprint plots, which indicate the most important inter­molecular contacts to be O⋯H/H⋯O (50.1%) and H⋯H (41.2%). The significant frequency of H⋯H and O⋯H/H⋯O inter­actions implies that van der Waals inter­actions and hydrogen bonding are critical in the crystal packing (Hathwar *et al.*, 2015 ▸) of the title complex (Fig. 4 ▸). We previously reported that *p*-xylyl doubly-bridged Zn^II^–cyclen, *p*-bis­(Zn^II^–cyclen), forms a characteristic helix-like supra­molecular structure (Ichimaru *et al.*, 2023 ▸). However, the packing of the title complex reported herein exhibits no specific supra­molecular structures.

## Synthesis and crystallization

The ligand *p*-bis­(cyclen), or 1,4-bis­((1,4,7,10-tetra­aza­cyclo­dodecan-1-yl)meth­yl)benzene, was synthesized as previously reported using an in-house lab method (Koike *et al.*, 1996 ▸). A solution of Cu(NO_3_)_2_·3H_2_O (484 mg, 2.0 mmol) in water (1.0 ml) was added dropwise to 20 ml of an ethano­lic solution of *p*-bis­(cyclen) (446 mg, 1.0 mmol). The reaction mixture was stirred for 30 min at 353 K and then filtered. The filtrate was allowed to stand 3 days at room temperature. Blue block-shaped crystals (600 mg) were obtained in 67% yield.

## Refinement

Crystal data, data collection and structure refinement details are summarized in Table 2 ▸. In the final cycles of refinement, 12 outliers were omitted.

## Supplementary Material

Crystal structure: contains datablock(s) I. DOI: 10.1107/S2414314623004625/zl4054sup1.cif

Structure factors: contains datablock(s) I. DOI: 10.1107/S2414314623004625/zl4054Isup2.hkl

CCDC reference: 2265479

Additional supporting information:  crystallographic information; 3D view; checkCIF report

## Figures and Tables

**Table 1:** Hydrogen-bond geometry (Å, °)

*D*—H⋯*A*	*D*—H	H⋯*A*	*D*⋯*A*	*D*—H⋯*A*
N2—H2⋯O7^i^	0.98 (3)	2.10 (3)	3.046 (7)	162 (6)
N3—H3⋯O10^ii^	0.99 (3)	2.17 (5)	3.032 (7)	145 (6)
N4—H4⋯O13^iii^	0.99 (3)	2.06 (5)	2.929 (8)	146 (6)
N6—H6⋯O13^iv^	0.97 (3)	1.95 (3)	2.900 (7)	167 (6)
N7—H7⋯O16^v^	0.97 (3)	2.00 (4)	2.934 (7)	160 (6)
N8—H8⋯O5	0.98 (3)	2.06 (3)	3.015 (7)	165 (6)
O4—H4*A*⋯O8	0.85 (3)	2.07 (6)	2.794 (8)	143 (7)
O14—H14*A*⋯O8	0.84 (2)	2.01 (3)	2.740 (7)	145 (5)
O14—H14*B*⋯O7	0.85 (3)	2.16 (3)	2.865 (7)	140 (5)
O15—H15*A*⋯O14	0.84 (3)	1.91 (3)	2.742 (7)	169 (8)
O15—H15*B*⋯O11	0.83 (3)	2.03 (4)	2.825 (7)	159 (8)
O16—H16*C*⋯O15	0.84 (3)	2.06 (5)	2.802 (7)	147 (7)
O16—H16*D*⋯O3^vi^	0.85 (3)	2.05 (5)	2.830 (7)	153 (9)

Symmetry codes: (i) 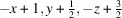 ; (ii) 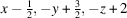 ; (iii) 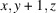 ; (iv) 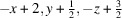 ; (v) 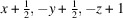 ; (vi) 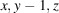 .

**Table 2:** Experimental details

Crystal data	
Chemical formula	[Cu_2_(NO_3_)(C_24_H_46_N_8_)(H_2_O)](NO_3_)_3_·3H_2_O
*M*_r_	893.87
Crystal system, space group	Orthorhombic, *P*2_1_2_1_2_1_
Temperature (K)	93
*a*, *b*, *c* (Å)	14.9788 (2), 15.3455 (2), 16.2948 (2)
*V* (Å^3^)	3745.48 (8)
*Z*	4
Radiation type	Cu *K*α
μ (mm^−1^)	2.14
Crystal size (mm)	0.55 × 0.45 × 0.17

Data collection	
Diffractometer	Rigaku XtaLAB Synergy-i
Absorption correction	Multi-scan (*CrysAlis PRO*; Rigaku OD, 2022 ▸)
*T*_min_, *T*_max_	0.505, 1.000
No. of measured, independent and observed [*I* > 2σ(*I*)] reflections	19219, 6617, 6227
*R*_int_	0.047
(sin θ/λ)_max_ (Å^−1^)	0.603

Refinement	
*R*[*F* ^2^ > 2σ(*F* ^2^)], *wR*(*F* ^2^), *S*	0.046, 0.139, 1.06
No. of reflections	6617
No. of parameters	530
No. of restraints	20
H-atom treatment	H atoms treated by a mixture of independent and constrained refinement
Δρ_max_, Δρ_min_ (e Å^−3^)	1.08, −0.53
Absolute structure	Refined as an inversion twin
Absolute structure parameter	0.36 (5)

Computer programs: *CrysAlis PRO* (Rigaku OD, 2022 ▸), *SHELXT2018/2* (Sheldrick, 2015*a* ▸), *SHELXL2018/3* (Sheldrick, 2015*b* ▸), and *OLEX2* (Dolomanov *et al.*, 2009 ▸).

## full crystallographic data

### Crystal data

**Table:**

[Cu_2_(NO_3_)(C_24_H_46_N_8_)(H_2_O)](NO_3_)_3_·3H_2_O	*D*_x_ = 1.585 Mg m^−^^3^
*M**_r_* = 893.87	Cu *K*α radiation, λ = 1.54184 Å
Orthorhombic, *P*2_1_2_1_2_1_	Cell parameters from 16388 reflections
*a* = 14.9788 (2) Å	θ = 2.7–68.2°
*b* = 15.3455 (2) Å	µ = 2.14 mm^−^^1^
*c* = 16.2948 (2) Å	*T* = 93 K
*V* = 3745.48 (8) Å^3^	Block, blue
*Z* = 4	0.55 × 0.45 × 0.17 mm
*F*(000) = 1872	

### Data collection

**Table:**

Rigaku XtaLAB Synergy-i diffractometer	6617 independent reflections
Radiation source: microfocus sealed X-ray tube, PhotonJet-i	6227 reflections with *I* > 2σ(*I*)
Multi-layer mirror optics monochromator	*R*_int_ = 0.047
Detector resolution: 10.0 pixels mm^-1^	θ_max_ = 68.4°, θ_min_ = 4.0°
ω scans	*h* = −16→18
Absorption correction: multi-scan (CrysAlisPro; Rigaku OD, 2022)	*k* = −18→18
*T*_min_ = 0.505, *T*_max_ = 1.000	*l* = −19→19
19219 measured reflections	

### Refinement

**Table:**

Refinement on *F*^2^	Hydrogen site location: mixed
Least-squares matrix: full	H atoms treated by a mixture of independent and constrained refinement
*R*[*F*^2^ > 2σ(*F*^2^)] = 0.046	*w* = 1/[σ^2^(*F*_o_^2^) + (0.0781*P*)^2^ + 6.1629*P*] where *P* = (*F*_o_^2^ + 2*F*_c_^2^)/3
*wR*(*F*^2^) = 0.139	(Δ/σ)_max_ < 0.001
*S* = 1.06	Δρ_max_ = 1.08 e Å^−^^3^
6617 reflections	Δρ_min_ = −0.53 e Å^−^^3^
530 parameters	Absolute structure: Refined as an inversion twin
20 restraints	Absolute structure parameter: 0.36 (5)

### Special details

**Table:**

Geometry. All esds (except the esd in the dihedral angle between two l.s. planes) are estimated using the full covariance matrix. The cell esds are taken into account individually in the estimation of esds in distances, angles and torsion angles; correlations between esds in cell parameters are only used when they are defined by crystal symmetry. An approximate (isotropic) treatment of cell esds is used for estimating esds involving l.s. planes.
Refinement. Refined as a 2-component inversion twin. All hydrogen atoms were located by a geometrical calculation, and were not refined.

### Fractional atomic coordinates and isotropic or equivalent isotropic displacement parameters (Å^2^)

**Table:**

	*x*	*y*	*z*	*U*_iso_*/*U*_eq_	
Cu1	0.61491 (6)	0.91479 (5)	1.03500 (5)	0.0204 (2)	
Cu2	0.88673 (5)	0.58988 (5)	0.45167 (5)	0.0199 (2)	
O1	0.5665 (3)	1.0063 (3)	0.9436 (4)	0.0473 (14)	
O2	0.4505 (4)	1.0249 (4)	1.0170 (3)	0.0501 (14)	
O3	0.4515 (4)	1.0675 (4)	0.8898 (3)	0.0472 (14)	
O4	0.9387 (4)	0.4801 (3)	0.5172 (3)	0.0427 (13)	
H4A	0.904 (4)	0.466 (6)	0.556 (4)	0.064*	
H4B	0.991 (2)	0.472 (7)	0.536 (5)	0.064*	
O5	0.6792 (4)	0.3885 (3)	0.4561 (3)	0.0452 (13)	
O6	0.7448 (3)	0.4371 (3)	0.5657 (4)	0.0419 (12)	
O7	0.6407 (3)	0.3400 (3)	0.5752 (3)	0.0403 (12)	
O8	0.9012 (3)	0.4147 (4)	0.6735 (4)	0.0533 (14)	
O9	1.0375 (4)	0.4523 (4)	0.6687 (4)	0.0524 (14)	
O10	0.9847 (4)	0.3927 (3)	0.7787 (3)	0.0423 (12)	
O11	0.7641 (3)	0.0649 (3)	0.8984 (3)	0.0365 (11)	
O12	0.8714 (4)	0.1596 (3)	0.8897 (3)	0.0414 (12)	
O13	0.8682 (4)	0.0673 (4)	0.9896 (3)	0.0584 (17)	
O14	0.7733 (4)	0.2886 (3)	0.6922 (3)	0.0397 (11)	
H14A	0.819 (3)	0.317 (4)	0.705 (4)	0.060*	
H14B	0.733 (4)	0.324 (4)	0.676 (3)	0.060*	
O15	0.6749 (3)	0.1819 (3)	0.7925 (3)	0.0331 (10)	
H15A	0.709 (4)	0.217 (4)	0.768 (5)	0.050*	
H15B	0.705 (4)	0.141 (4)	0.812 (5)	0.050*	
O16	0.5260 (3)	0.0921 (4)	0.7318 (3)	0.0389 (11)	
H16C	0.570 (4)	0.125 (5)	0.730 (5)	0.058*	
H16D	0.520 (5)	0.076 (6)	0.781 (2)	0.058*	
N1	0.6821 (3)	0.8349 (3)	0.9539 (3)	0.0208 (10)	
N2	0.5198 (3)	0.8225 (3)	1.0385 (3)	0.0233 (10)	
H2	0.462 (3)	0.833 (4)	1.012 (4)	0.028*	
N3	0.5738 (4)	0.9472 (3)	1.1495 (3)	0.0260 (11)	
H3	0.547 (4)	1.006 (2)	1.148 (5)	0.031*	
N4	0.7366 (3)	0.9556 (3)	1.0708 (3)	0.0230 (10)	
H4	0.758 (4)	1.007 (3)	1.040 (4)	0.028*	
N5	0.8166 (3)	0.6661 (3)	0.5318 (3)	0.0190 (10)	
N6	0.9851 (3)	0.6791 (3)	0.4614 (3)	0.0242 (11)	
H6	1.038 (3)	0.649 (4)	0.482 (4)	0.029*	
N7	0.9393 (4)	0.5610 (3)	0.3409 (3)	0.0268 (11)	
H7	0.957 (5)	0.503 (2)	0.324 (5)	0.032*	
N8	0.7683 (4)	0.5560 (3)	0.4049 (3)	0.0249 (11)	
H8	0.740 (4)	0.506 (3)	0.432 (4)	0.030*	
N9	0.4899 (4)	1.0309 (4)	0.9475 (4)	0.0314 (12)	
N10	0.6877 (3)	0.3903 (3)	0.5318 (4)	0.0310 (12)	
N11	0.9758 (4)	0.4207 (4)	0.7080 (3)	0.0295 (12)	
N12	0.8339 (4)	0.0990 (4)	0.9262 (3)	0.0322 (12)	
C1	0.6497 (4)	0.7470 (4)	0.9795 (4)	0.0226 (13)	
H1A	0.676124	0.731200	1.033150	0.027*	
H1B	0.668615	0.702879	0.938650	0.027*	
C2	0.5475 (4)	0.7478 (4)	0.9861 (4)	0.0263 (13)	
H2A	0.520778	0.753505	0.930819	0.032*	
H2B	0.526427	0.692444	1.010611	0.032*	
C3	0.5102 (4)	0.8018 (5)	1.1271 (4)	0.0287 (14)	
H3A	0.563534	0.770084	1.147014	0.034*	
H3B	0.457054	0.764620	1.136172	0.034*	
C4	0.5000 (4)	0.8879 (5)	1.1730 (4)	0.0326 (15)	
H4C	0.441937	0.914948	1.159122	0.039*	
H4D	0.501488	0.877340	1.232907	0.039*	
C5	0.6569 (4)	0.9380 (4)	1.1996 (4)	0.0300 (14)	
H5A	0.672719	0.875709	1.205537	0.036*	
H5B	0.647460	0.962771	1.255041	0.036*	
C6	0.7307 (5)	0.9859 (4)	1.1568 (4)	0.0305 (14)	
H6A	0.718734	1.049316	1.158026	0.037*	
H6B	0.788020	0.974994	1.185217	0.037*	
C7	0.7924 (4)	0.8762 (4)	1.0597 (4)	0.0248 (12)	
H7A	0.773451	0.830039	1.098373	0.030*	
H7B	0.856026	0.889860	1.070183	0.030*	
C8	0.7799 (4)	0.8462 (4)	0.9722 (4)	0.0229 (12)	
H8A	0.805916	0.889639	0.934198	0.027*	
H8B	0.811424	0.790146	0.963839	0.027*	
C9	0.6623 (4)	0.8556 (4)	0.8665 (3)	0.0233 (12)	
H9A	0.680441	0.916610	0.855953	0.028*	
H9B	0.596932	0.852083	0.858382	0.028*	
C10	0.7071 (4)	0.7977 (4)	0.8033 (3)	0.0199 (12)	
C11	0.6632 (4)	0.7253 (4)	0.7716 (3)	0.0230 (12)	
H11	0.605083	0.711175	0.790669	0.028*	
C12	0.7033 (4)	0.6736 (4)	0.7124 (3)	0.0218 (12)	
H12	0.672317	0.624829	0.690649	0.026*	
C13	0.7893 (4)	0.6932 (4)	0.6846 (3)	0.0189 (11)	
C14	0.8336 (4)	0.7642 (4)	0.7162 (3)	0.0225 (12)	
H14	0.892253	0.777662	0.697892	0.027*	
C15	0.7926 (4)	0.8162 (4)	0.7747 (3)	0.0226 (12)	
H15	0.823535	0.865472	0.795792	0.027*	
C16	0.8321 (4)	0.6374 (4)	0.6192 (3)	0.0218 (12)	
H16A	0.897222	0.635855	0.629262	0.026*	
H16B	0.809426	0.577091	0.625190	0.026*	
C17	0.8537 (4)	0.7544 (4)	0.5142 (3)	0.0200 (12)	
H17A	0.832624	0.796033	0.556322	0.024*	
H17B	0.831839	0.774598	0.460073	0.024*	
C18	0.9549 (4)	0.7524 (4)	0.5138 (4)	0.0246 (13)	
H18A	0.978530	0.808125	0.492194	0.030*	
H18B	0.977529	0.744618	0.570468	0.030*	
C19	1.0034 (4)	0.7033 (5)	0.3757 (4)	0.0303 (15)	
H19A	1.057911	0.739556	0.372507	0.036*	
H19B	0.952808	0.737113	0.352941	0.036*	
C20	1.0160 (5)	0.6202 (5)	0.3276 (4)	0.0347 (16)	
H20A	1.021283	0.633910	0.268444	0.042*	
H20B	1.071770	0.591116	0.345272	0.042*	
C21	0.8636 (4)	0.5745 (4)	0.2841 (3)	0.0277 (13)	
H21A	0.878321	0.550350	0.229443	0.033*	
H21B	0.851715	0.637622	0.277868	0.033*	
C22	0.7817 (5)	0.5294 (4)	0.3182 (4)	0.0293 (14)	
H22A	0.728652	0.545398	0.285284	0.035*	
H22B	0.789563	0.465434	0.315060	0.035*	
C23	0.7142 (4)	0.6365 (4)	0.4163 (3)	0.0245 (13)	
H23A	0.651252	0.625843	0.400546	0.029*	
H23B	0.737992	0.684399	0.382023	0.029*	
C24	0.7204 (4)	0.6599 (4)	0.5068 (3)	0.0230 (13)	
H24A	0.690306	0.716319	0.516781	0.028*	
H24B	0.690063	0.614774	0.540082	0.028*	

### Atomic displacement parameters (Å^2^)

**Table:**

	*U*^11^	*U*^22^	*U*^33^	*U*^12^	*U*^13^	*U*^23^
Cu1	0.0235 (4)	0.0245 (4)	0.0131 (4)	0.0044 (4)	0.0004 (3)	−0.0001 (3)
Cu2	0.0227 (4)	0.0239 (4)	0.0131 (4)	0.0004 (4)	0.0014 (3)	−0.0011 (3)
O1	0.033 (3)	0.054 (3)	0.055 (4)	0.014 (2)	0.004 (2)	0.025 (3)
O2	0.049 (3)	0.066 (3)	0.035 (3)	0.014 (3)	0.012 (2)	0.011 (3)
O3	0.044 (3)	0.064 (4)	0.034 (3)	0.028 (3)	−0.002 (2)	0.016 (3)
O4	0.065 (3)	0.038 (3)	0.025 (3)	0.019 (3)	−0.002 (2)	0.004 (2)
O5	0.048 (3)	0.044 (3)	0.043 (3)	−0.010 (2)	−0.016 (3)	0.013 (2)
O6	0.033 (2)	0.037 (2)	0.056 (3)	−0.004 (2)	−0.011 (2)	−0.006 (2)
O7	0.036 (3)	0.045 (3)	0.039 (3)	−0.016 (2)	−0.006 (2)	0.005 (2)
O8	0.035 (3)	0.068 (3)	0.056 (3)	−0.012 (3)	−0.013 (3)	0.015 (3)
O9	0.038 (3)	0.059 (3)	0.061 (4)	−0.012 (3)	0.004 (3)	0.011 (3)
O10	0.055 (3)	0.043 (3)	0.029 (3)	0.000 (2)	−0.013 (2)	0.006 (2)
O11	0.032 (2)	0.044 (3)	0.033 (3)	−0.014 (2)	−0.009 (2)	0.007 (2)
O12	0.052 (3)	0.043 (3)	0.030 (2)	−0.019 (2)	−0.001 (2)	0.005 (2)
O13	0.057 (3)	0.084 (4)	0.035 (3)	−0.033 (3)	−0.021 (3)	0.023 (3)
O14	0.045 (3)	0.038 (2)	0.036 (3)	−0.002 (2)	0.010 (2)	0.002 (2)
O15	0.030 (2)	0.035 (2)	0.034 (3)	−0.001 (2)	−0.002 (2)	0.004 (2)
O16	0.036 (3)	0.047 (3)	0.033 (2)	−0.012 (2)	0.000 (2)	−0.004 (2)
N1	0.023 (2)	0.027 (2)	0.013 (2)	0.005 (2)	0.0003 (19)	0.0019 (19)
N2	0.023 (2)	0.035 (3)	0.013 (2)	0.007 (2)	−0.005 (2)	−0.002 (2)
N3	0.028 (3)	0.033 (3)	0.017 (2)	0.007 (2)	0.002 (2)	0.000 (2)
N4	0.029 (3)	0.026 (2)	0.014 (2)	0.001 (2)	−0.001 (2)	−0.001 (2)
N5	0.019 (2)	0.028 (2)	0.010 (2)	−0.0010 (19)	0.0015 (18)	−0.0020 (19)
N6	0.024 (2)	0.031 (3)	0.017 (2)	0.004 (2)	0.003 (2)	0.002 (2)
N7	0.033 (3)	0.030 (3)	0.017 (2)	0.005 (2)	0.005 (2)	−0.001 (2)
N8	0.032 (3)	0.027 (2)	0.016 (2)	−0.005 (2)	0.000 (2)	0.003 (2)
N9	0.033 (3)	0.028 (3)	0.034 (3)	−0.001 (2)	−0.006 (2)	0.006 (2)
N10	0.022 (2)	0.025 (3)	0.046 (3)	−0.002 (2)	−0.002 (2)	−0.002 (2)
N11	0.027 (3)	0.033 (3)	0.029 (3)	0.002 (2)	−0.006 (2)	−0.004 (2)
N12	0.035 (3)	0.038 (3)	0.023 (3)	−0.007 (3)	0.000 (2)	0.000 (2)
C1	0.025 (3)	0.024 (3)	0.019 (3)	0.007 (2)	0.002 (2)	0.001 (2)
C2	0.026 (3)	0.032 (3)	0.021 (3)	0.001 (3)	−0.005 (2)	−0.003 (3)
C3	0.028 (3)	0.037 (4)	0.021 (3)	−0.001 (3)	0.004 (3)	0.003 (3)
C4	0.035 (4)	0.049 (4)	0.014 (3)	0.003 (3)	0.006 (3)	0.002 (3)
C5	0.033 (3)	0.039 (3)	0.018 (3)	0.001 (3)	0.002 (3)	0.000 (2)
C6	0.040 (4)	0.035 (3)	0.017 (3)	−0.001 (3)	0.001 (3)	−0.006 (3)
C7	0.027 (3)	0.033 (3)	0.015 (3)	0.004 (2)	−0.003 (2)	0.000 (2)
C8	0.019 (3)	0.031 (3)	0.019 (3)	0.010 (2)	−0.002 (2)	0.000 (2)
C9	0.032 (3)	0.028 (3)	0.010 (3)	0.008 (3)	−0.001 (2)	0.003 (2)
C10	0.028 (3)	0.023 (3)	0.009 (2)	0.006 (2)	−0.006 (2)	0.003 (2)
C11	0.021 (3)	0.034 (3)	0.015 (3)	−0.001 (2)	−0.001 (2)	0.005 (2)
C12	0.024 (3)	0.027 (3)	0.015 (3)	−0.002 (2)	−0.001 (2)	−0.002 (2)
C13	0.024 (3)	0.023 (3)	0.010 (2)	0.000 (2)	−0.003 (2)	0.003 (2)
C14	0.027 (3)	0.023 (3)	0.018 (3)	0.001 (2)	−0.001 (2)	0.000 (2)
C15	0.028 (3)	0.030 (3)	0.010 (2)	−0.003 (2)	0.000 (2)	0.000 (2)
C16	0.027 (3)	0.025 (3)	0.013 (3)	0.005 (2)	−0.003 (2)	−0.002 (2)
C17	0.027 (3)	0.021 (3)	0.012 (3)	−0.001 (2)	−0.001 (2)	0.002 (2)
C18	0.028 (3)	0.026 (3)	0.020 (3)	−0.003 (2)	0.003 (2)	−0.004 (2)
C19	0.029 (3)	0.041 (4)	0.021 (3)	−0.007 (3)	0.006 (3)	0.002 (3)
C20	0.033 (3)	0.048 (4)	0.023 (3)	0.008 (3)	0.011 (3)	0.001 (3)
C21	0.038 (4)	0.036 (3)	0.010 (2)	0.005 (3)	0.001 (2)	0.001 (2)
C22	0.045 (4)	0.033 (3)	0.010 (3)	0.000 (3)	−0.004 (3)	−0.006 (2)
C23	0.028 (3)	0.036 (3)	0.009 (3)	0.001 (3)	0.000 (2)	−0.001 (2)
C24	0.016 (3)	0.036 (3)	0.016 (3)	0.000 (2)	0.001 (2)	−0.002 (2)

### Geometric parameters (Å, º)

**Table:**

Cu1—O1	2.170 (5)	C1—H1B	0.9900
Cu1—N1	2.065 (5)	C1—C2	1.535 (9)
Cu1—N2	2.011 (5)	C2—H2A	0.9900
Cu1—N3	2.026 (5)	C2—H2B	0.9900
Cu1—N4	2.013 (5)	C3—H3A	0.9900
Cu2—O4	2.141 (5)	C3—H3B	0.9900
Cu2—N5	2.044 (5)	C3—C4	1.526 (9)
Cu2—N6	2.017 (5)	C4—H4C	0.9900
Cu2—N7	2.019 (5)	C4—H4D	0.9900
Cu2—N8	2.000 (5)	C5—H5A	0.9900
O1—N9	1.211 (7)	C5—H5B	0.9900
O2—N9	1.281 (8)	C5—C6	1.499 (9)
O3—N9	1.237 (7)	C6—H6A	0.9900
O4—H4A	0.85 (3)	C6—H6B	0.9900
O4—H4B	0.85 (3)	C7—H7A	0.9900
O5—N10	1.241 (8)	C7—H7B	0.9900
O6—N10	1.246 (7)	C7—C8	1.510 (8)
O7—N10	1.261 (7)	C8—H8A	0.9900
O8—N11	1.254 (7)	C8—H8B	0.9900
O9—N11	1.225 (8)	C9—H9A	0.9900
O10—N11	1.236 (7)	C9—H9B	0.9900
O11—N12	1.255 (7)	C9—C10	1.518 (8)
O12—N12	1.238 (7)	C10—C11	1.390 (8)
O13—N12	1.252 (7)	C10—C15	1.392 (9)
O14—H14A	0.84 (2)	C11—H11	0.9500
O14—H14B	0.85 (3)	C11—C12	1.386 (8)
O15—H15A	0.84 (3)	C12—H12	0.9500
O15—H15B	0.83 (3)	C12—C13	1.397 (8)
O16—H16C	0.84 (3)	C13—C14	1.377 (8)
O16—H16D	0.85 (3)	C13—C16	1.511 (8)
N1—C1	1.492 (7)	C14—H14	0.9500
N1—C8	1.505 (7)	C14—C15	1.387 (8)
N1—C9	1.489 (7)	C15—H15	0.9500
N2—H2	0.98 (3)	C16—H16A	0.9900
N2—C2	1.488 (8)	C16—H16B	0.9900
N2—C3	1.486 (7)	C17—H17A	0.9900
N3—H3	0.99 (3)	C17—H17B	0.9900
N3—C4	1.482 (8)	C17—C18	1.517 (9)
N3—C5	1.496 (8)	C18—H18A	0.9900
N4—H4	0.99 (3)	C18—H18B	0.9900
N4—C6	1.478 (8)	C19—H19A	0.9900
N4—C7	1.490 (7)	C19—H19B	0.9900
N5—C16	1.508 (7)	C19—C20	1.508 (10)
N5—C17	1.491 (7)	C20—H20A	0.9900
N5—C24	1.501 (7)	C20—H20B	0.9900
N6—H6	0.97 (3)	C21—H21A	0.9900
N6—C18	1.484 (8)	C21—H21B	0.9900
N6—C19	1.471 (7)	C21—C22	1.514 (9)
N7—H7	0.97 (3)	C22—H22A	0.9900
N7—C20	1.480 (9)	C22—H22B	0.9900
N7—C21	1.479 (8)	C23—H23A	0.9900
N8—H8	0.98 (3)	C23—H23B	0.9900
N8—C22	1.484 (7)	C23—C24	1.521 (8)
N8—C23	1.489 (8)	C24—H24A	0.9900
C1—H1A	0.9900	C24—H24B	0.9900
			
N1—Cu1—O1	96.2 (2)	N3—C4—H4D	109.8
N2—Cu1—O1	103.8 (2)	C3—C4—H4C	109.8
N2—Cu1—N1	86.85 (19)	C3—C4—H4D	109.8
N2—Cu1—N3	86.1 (2)	H4C—C4—H4D	108.3
N2—Cu1—N4	148.5 (2)	N3—C5—H5A	110.0
N3—Cu1—O1	111.8 (2)	N3—C5—H5B	110.0
N3—Cu1—N1	152.0 (2)	N3—C5—C6	108.3 (5)
N4—Cu1—O1	107.4 (2)	H5A—C5—H5B	108.4
N4—Cu1—N1	85.93 (19)	C6—C5—H5A	110.0
N4—Cu1—N3	86.1 (2)	C6—C5—H5B	110.0
N5—Cu2—O4	108.55 (19)	N4—C6—C5	109.3 (5)
N6—Cu2—O4	103.2 (2)	N4—C6—H6A	109.8
N6—Cu2—N5	86.4 (2)	N4—C6—H6B	109.8
N6—Cu2—N7	86.2 (2)	C5—C6—H6A	109.8
N7—Cu2—O4	97.5 (2)	C5—C6—H6B	109.8
N7—Cu2—N5	153.9 (2)	H6A—C6—H6B	108.3
N8—Cu2—O4	108.0 (2)	N4—C7—H7A	110.3
N8—Cu2—N5	86.4 (2)	N4—C7—H7B	110.3
N8—Cu2—N6	148.7 (2)	N4—C7—C8	107.2 (5)
N8—Cu2—N7	87.0 (2)	H7A—C7—H7B	108.5
N9—O1—Cu1	118.9 (4)	C8—C7—H7A	110.3
Cu2—O4—H4A	110 (6)	C8—C7—H7B	110.3
Cu2—O4—H4B	129 (7)	N1—C8—C7	110.1 (5)
H4A—O4—H4B	106 (4)	N1—C8—H8A	109.7
H14A—O14—H14B	109 (4)	N1—C8—H8B	109.7
H15A—O15—H15B	110 (4)	C7—C8—H8A	109.7
H16C—O16—H16D	107 (4)	C7—C8—H8B	109.7
C1—N1—Cu1	101.5 (3)	H8A—C8—H8B	108.2
C1—N1—C8	111.4 (4)	N1—C9—H9A	108.3
C8—N1—Cu1	106.2 (3)	N1—C9—H9B	108.3
C9—N1—Cu1	112.8 (3)	N1—C9—C10	115.9 (4)
C9—N1—C1	113.3 (5)	H9A—C9—H9B	107.4
C9—N1—C8	111.0 (5)	C10—C9—H9A	108.3
Cu1—N2—H2	120 (4)	C10—C9—H9B	108.3
C2—N2—Cu1	109.2 (4)	C11—C10—C9	120.7 (5)
C2—N2—H2	97 (4)	C11—C10—C15	118.3 (5)
C3—N2—Cu1	104.3 (4)	C15—C10—C9	120.9 (5)
C3—N2—H2	112 (4)	C10—C11—H11	119.6
C3—N2—C2	114.8 (5)	C12—C11—C10	120.7 (5)
Cu1—N3—H3	109 (4)	C12—C11—H11	119.6
C4—N3—Cu1	108.3 (4)	C11—C12—H12	119.9
C4—N3—H3	105 (4)	C11—C12—C13	120.1 (5)
C4—N3—C5	115.0 (5)	C13—C12—H12	119.9
C5—N3—Cu1	103.1 (4)	C12—C13—C16	119.9 (5)
C5—N3—H3	116 (4)	C14—C13—C12	119.6 (5)
Cu1—N4—H4	113 (4)	C14—C13—C16	120.5 (5)
C6—N4—Cu1	108.6 (4)	C13—C14—H14	120.0
C6—N4—H4	105 (4)	C13—C14—C15	119.9 (6)
C6—N4—C7	113.9 (5)	C15—C14—H14	120.0
C7—N4—Cu1	102.6 (4)	C10—C15—H15	119.4
C7—N4—H4	114 (4)	C14—C15—C10	121.3 (6)
C16—N5—Cu2	110.9 (3)	C14—C15—H15	119.4
C17—N5—Cu2	101.8 (3)	N5—C16—C13	115.8 (4)
C17—N5—C16	113.0 (4)	N5—C16—H16A	108.3
C17—N5—C24	111.3 (5)	N5—C16—H16B	108.3
C24—N5—Cu2	106.4 (3)	C13—C16—H16A	108.3
C24—N5—C16	112.6 (4)	C13—C16—H16B	108.3
Cu2—N6—H6	107 (4)	H16A—C16—H16B	107.4
C18—N6—Cu2	109.7 (4)	N5—C17—H17A	109.5
C18—N6—H6	114 (4)	N5—C17—H17B	109.5
C19—N6—Cu2	103.5 (4)	N5—C17—C18	110.8 (5)
C19—N6—H6	107 (4)	H17A—C17—H17B	108.1
C19—N6—C18	114.3 (5)	C18—C17—H17A	109.5
Cu2—N7—H7	124 (5)	C18—C17—H17B	109.5
C20—N7—Cu2	107.4 (4)	N6—C18—C17	108.7 (5)
C20—N7—H7	108 (4)	N6—C18—H18A	109.9
C21—N7—Cu2	103.2 (4)	N6—C18—H18B	109.9
C21—N7—H7	100 (4)	C17—C18—H18A	109.9
C21—N7—C20	114.7 (5)	C17—C18—H18B	109.9
Cu2—N8—H8	114 (4)	H18A—C18—H18B	108.3
C22—N8—Cu2	108.3 (4)	N6—C19—H19A	110.2
C22—N8—H8	105 (4)	N6—C19—H19B	110.2
C22—N8—C23	114.9 (5)	N6—C19—C20	107.6 (6)
C23—N8—Cu2	102.7 (4)	H19A—C19—H19B	108.5
C23—N8—H8	111 (4)	C20—C19—H19A	110.2
O1—N9—O2	117.4 (6)	C20—C19—H19B	110.2
O1—N9—O3	122.9 (6)	N7—C20—C19	110.2 (5)
O3—N9—O2	119.4 (5)	N7—C20—H20A	109.6
O5—N10—O6	121.6 (6)	N7—C20—H20B	109.6
O5—N10—O7	119.1 (5)	C19—C20—H20A	109.6
O6—N10—O7	119.2 (6)	C19—C20—H20B	109.6
O9—N11—O8	117.8 (6)	H20A—C20—H20B	108.1
O9—N11—O10	123.0 (6)	N7—C21—H21A	109.9
O10—N11—O8	119.2 (6)	N7—C21—H21B	109.9
O12—N12—O11	121.1 (5)	N7—C21—C22	109.1 (5)
O12—N12—O13	120.2 (6)	H21A—C21—H21B	108.3
O13—N12—O11	118.6 (5)	C22—C21—H21A	109.9
N1—C1—H1A	109.7	C22—C21—H21B	109.9
N1—C1—H1B	109.7	N8—C22—C21	109.5 (5)
N1—C1—C2	109.7 (5)	N8—C22—H22A	109.8
H1A—C1—H1B	108.2	N8—C22—H22B	109.8
C2—C1—H1A	109.7	C21—C22—H22A	109.8
C2—C1—H1B	109.7	C21—C22—H22B	109.8
N2—C2—C1	109.0 (5)	H22A—C22—H22B	108.2
N2—C2—H2A	109.9	N8—C23—H23A	110.4
N2—C2—H2B	109.9	N8—C23—H23B	110.4
C1—C2—H2A	109.9	N8—C23—C24	106.5 (5)
C1—C2—H2B	109.9	H23A—C23—H23B	108.6
H2A—C2—H2B	108.3	C24—C23—H23A	110.4
N2—C3—H3A	110.2	C24—C23—H23B	110.4
N2—C3—H3B	110.2	N5—C24—C23	109.8 (5)
N2—C3—C4	107.5 (5)	N5—C24—H24A	109.7
H3A—C3—H3B	108.5	N5—C24—H24B	109.7
C4—C3—H3A	110.2	C23—C24—H24A	109.7
C4—C3—H3B	110.2	C23—C24—H24B	109.7
N3—C4—C3	109.3 (5)	H24A—C24—H24B	108.2
N3—C4—H4C	109.8		
			
Cu1—O1—N9—O2	21.7 (8)	C3—N2—C2—C1	−91.8 (6)
Cu1—O1—N9—O3	−164.3 (5)	C4—N3—C5—C6	166.9 (5)
Cu1—N1—C1—C2	49.1 (5)	C5—N3—C4—C3	−87.5 (6)
Cu1—N1—C8—C7	24.5 (5)	C6—N4—C7—C8	171.4 (5)
Cu1—N1—C9—C10	−178.2 (4)	C7—N4—C6—C5	−85.7 (6)
Cu1—N2—C2—C1	24.8 (6)	C8—N1—C1—C2	161.9 (5)
Cu1—N2—C3—C4	49.5 (5)	C8—N1—C9—C10	62.7 (6)
Cu1—N3—C4—C3	27.1 (6)	C9—N1—C1—C2	−72.1 (6)
Cu1—N3—C5—C6	49.3 (5)	C9—N1—C8—C7	147.5 (5)
Cu1—N4—C6—C5	27.9 (6)	C9—C10—C11—C12	178.5 (5)
Cu1—N4—C7—C8	54.2 (5)	C9—C10—C15—C14	−179.2 (5)
Cu2—N5—C16—C13	175.5 (4)	C10—C11—C12—C13	0.9 (8)
Cu2—N5—C17—C18	−49.1 (5)	C11—C10—C15—C14	0.0 (8)
Cu2—N5—C24—C23	−24.9 (6)	C11—C12—C13—C14	−0.3 (8)
Cu2—N6—C18—C17	−22.2 (6)	C11—C12—C13—C16	−179.1 (5)
Cu2—N6—C19—C20	−50.2 (5)	C12—C13—C14—C15	−0.5 (8)
Cu2—N7—C20—C19	−26.4 (6)	C12—C13—C16—N5	90.8 (7)
Cu2—N7—C21—C22	−47.7 (5)	C13—C14—C15—C10	0.6 (8)
Cu2—N8—C22—C21	−26.3 (6)	C14—C13—C16—N5	−88.0 (6)
Cu2—N8—C23—C24	−54.1 (5)	C15—C10—C11—C12	−0.7 (8)
N1—C1—C2—N2	−51.4 (6)	C16—N5—C17—C18	70.0 (6)
N1—C9—C10—C11	94.2 (6)	C16—N5—C24—C23	−146.6 (5)
N1—C9—C10—C15	−86.7 (7)	C16—C13—C14—C15	178.3 (5)
N2—C3—C4—N3	−52.0 (7)	C17—N5—C16—C13	61.9 (7)
N3—C5—C6—N4	−52.8 (7)	C17—N5—C24—C23	85.3 (6)
N4—C7—C8—N1	−53.8 (6)	C18—N6—C19—C20	−169.5 (5)
N5—C17—C18—N6	49.2 (6)	C19—N6—C18—C17	93.5 (6)
N6—C19—C20—N7	52.6 (7)	C20—N7—C21—C22	−164.2 (5)
N7—C21—C22—N8	50.9 (7)	C21—N7—C20—C19	87.7 (6)
N8—C23—C24—N5	53.9 (6)	C22—N8—C23—C24	−171.5 (5)
C1—N1—C8—C7	−85.3 (6)	C23—N8—C22—C21	87.8 (6)
C1—N1—C9—C10	−63.5 (7)	C24—N5—C16—C13	−65.3 (6)
C2—N2—C3—C4	168.9 (5)	C24—N5—C17—C18	−162.1 (5)

### Hydrogen-bond geometry (Å, º)

**Table:**

*D*—H···*A*	*D*—H	H···*A*	*D*···*A*	*D*—H···*A*
N2—H2···O7^i^	0.98 (3)	2.10 (3)	3.046 (7)	162 (6)
N3—H3···O10^ii^	0.99 (3)	2.17 (5)	3.032 (7)	145 (6)
N4—H4···O13^iii^	0.99 (3)	2.06 (5)	2.929 (8)	146 (6)
N6—H6···O13^iv^	0.97 (3)	1.95 (3)	2.900 (7)	167 (6)
N7—H7···O16^v^	0.97 (3)	2.00 (4)	2.934 (7)	160 (6)
N8—H8···O5	0.98 (3)	2.06 (3)	3.015 (7)	165 (6)
O4—H4*A*···O8	0.85 (3)	2.07 (6)	2.794 (8)	143 (7)
O14—H14*A*···O8	0.84 (2)	2.01 (3)	2.740 (7)	145 (5)
O14—H14*B*···O7	0.85 (3)	2.16 (3)	2.865 (7)	140 (5)
O15—H15*A*···O14	0.84 (3)	1.91 (3)	2.742 (7)	169 (8)
O15—H15*B*···O11	0.83 (3)	2.03 (4)	2.825 (7)	159 (8)
O16—H16*C*···O15	0.84 (3)	2.06 (5)	2.802 (7)	147 (7)
O16—H16*D*···O3^vi^	0.85 (3)	2.05 (5)	2.830 (7)	153 (9)

Symmetry codes: (i) −*x*+1, *y*+1/2, −*z*+3/2; (ii) *x*−1/2, −*y*+3/2, −*z*+2; (iii) *x*, *y*+1, *z*; (iv) −*x*+2, *y*+1/2, −*z*+3/2; (v) *x*+1/2, −*y*+1/2, −*z*+1; (vi) *x*, *y*−1, *z*.
